# Impact of a Combination of UV-C Irradiation and Peracetic Acid Spray Treatment on *Brochothrix thermosphacta* and *Yersinia enterocolitica* Contaminated Pork

**DOI:** 10.3390/foods10020204

**Published:** 2021-01-20

**Authors:** Valerie Koller, Diana Seinige, Julia Saathoff, Corinna Kehrenberg, Carsten Krischek

**Affiliations:** 1 Institute of Food Quality and Food Safety, University of Veterinary Medicine Hannover, Foundation, Bischofsholer Damm 15, 30173 Hannover, Germany; Valerie.Koller@tiho-hannover.de (V.K.); Julia.Saathoff@tiho-hannover.de (J.S.); 2 Lower Saxony State Office for Consumer Protection and Food Safety, Röverskamp 5, 26203 Wardenburg, Germany; Diana.Seinige@gmx.de; 3 Institute for Veterinary Food Science, Justus-Liebig-University Giessen, Frankfurter Str. 92, 35392 Giessen, Germany; Corinna.Kehrenberg@vetmed.uni-giessen.de

**Keywords:** *Yersinia enterocolitica*, *Brochothrix thermosphacta*, UV-C, peracetic acid, pork, meat quality

## Abstract

Efficient ways of decontamination are needed to minimize the risk of infections with *Yersinia (Y.) enterocolitica*, which causes gastrointestinal diseases in humans, and to reduce the numbers of *Brochothrix (B.) thermosphacta* to extend the shelf-life of meat. While many studies have focused on a single treatment of peracetic acid (PAA) or UV-C-irradiation, there are no studies about a combined treatment on meat. Therefore, in the present study, pork was inoculated with either *Y. enterocolitica* or *B. thermosphacta*, and was treated with a combination of 2040 mJ/cm^2^ UV-C irradiation followed by a 2000 ppm PAA spray treatment (30 s). Samples were packed under modified atmosphere and stored for 1, 7, or 14 days. The samples were examined for *Y. enterocolitica* and *B. thermosphacta* content, chemical and sensory effects, and meat quality parameters. For *Y. enterocolitica*, a significant reduction of up to 2.16 log_10_ cfu/cm^2^ meat and for *B. thermosphacta*, up to 2.37 log_10_ cfu/cm^2^ meat was seen on day 14 after UV-C/PAA treatment compared to the untreated controls.

## 1. Introduction

The consumption of meat—especially pork and poultry—is increasing worldwide [1]. Meat and meat products can be cross-contaminated with microorganisms during slaughter or meat processing. This results in a risk for the consumer from pathogenic bacteria such as *Yersinia* (*Y.*) *enterocolitica* and a reduced shelf-life, especially after contamination with spoilage bacteria such as *Brochothrix* (*B.*) *thermosphacta*.

Yersiniosis is one of the most important foodborne diseases worldwide. In 2018, it was reported as the third most frequently occurring bacterial zoonosis in the EU, with 6699 confirmed cases [2]. Pigs are an important reservoir for *Y. enterocolitica* and, therefore, pork might pose a risk for *Yersinia* infections [3,4]. Pigs are usually asymptomatic carriers, and consequently the bacteria are frequently isolated from pig carcasses [3]. The microorganisms may contaminate other carcasses and meat during further processing and, due to their psychrotrophic properties, can multiply during chilled storage. After evisceration, *Y. enterocolitica* 4/O:3 can be found on 40% of the pig carcasses [5]. In 2018, *Yersinia* spp. were detected in 5% of the meat and meat products from pigs sampled in the EU [2]. Besides contamination with pathogenic bacteria, contamination with spoilage bacteria such as *B. thermosphacta* should also be considered. Today, the requirements for the quality of fresh meat are getting higher and the transportation distances are getting longer, so it is necessary to reduce the bacterial count on fresh meat in order to extend its shelf life. *B. thermosphacta* is an important spoilage bacterium in packaged meat. It can be detected in almost every examined meat and meat product [6]. Like *Y. enterocolitica*, it is a psychrotrophic species that can be found in the environment of slaughterhouses, on carcasses, in the cutting area and also on workers [7].

To minimize the frequency of contaminated meat, several preservation methods might be applied, which are mainly divided into physical, chemical, and biological methods. One physical method to reduce the bacterial load on food is ultraviolet (UV) light [8]. Previous studies have shown that UV-C light is effective against bacteria such as *Salmonella* (*S.*) spp., *Escherichia (E.) coli*, *B. thermosphacta*, and *Y. enterocolitica* with the greatest antimicrobial effect at a wavelength of 254 nm [8,9,10,11,12]. The advantage of UV-C treatment is that no residues are left on the food [13], and it is therefore considered as safe for humans. However, it can lead to changes in meat quality such as the color of meat [14,15,16]. When bacteria are UV-C irradiated, high energy photons damage the DNA by shifting electrons that break the DNA bonds. Cross-linkings between adjacent DNA bases of the same strand are built, and the bacterial transcription and replication is stopped. The quantity of the UV-C dose is proportional to the cross-linking effects [9]. Major damages can be lethal for the bacterium, while small damages can be repaired simultaneously to the UV irradiation or directly afterwards with the enzyme photolyase, which is activated by light with a wavelength of 310–330 to 480 nm. This process is known as photoreactivation [10,17,18].

In contrast, peracetic acid (PAA) treatment is a chemical preservation method to diminish the bacterial count on meat. It can be applied as a dipping or spray treatment. In the USA, it is already being used to decontaminate chicken carcasses in chiller tanks. And in New Zealand, it is approved for rinsing bobby calf veal [19]. It is considered safe for humans in concentrations of up to 2000 ppm PAA for spray applications, because PAA dissociates to innocuous acetic acid, water, and oxygen [20]. PAA has a wide spectrum of antimicrobial activity already at low concentrations. It is considered effective in heterogeneous organic matter, with a low dependence on pH and temperature, hardly affected by protein residues, and effective even at short contact times [21,22,23]. But PAA can also affect meat quality such as the color of meat [24,25,26].

There are several studies published that presented results after treatment of meat with either PAA [27,28,29], or UV-C light [11,14,30,31], which showed a reduction of various bacteria through the treatment. However, there are no studies available that present results after a combined treatment of meat. Since UV-C irradiation mainly damages nucleic acids and PAA mainly attacks cell walls and membranes, the damage that occurs in a combined treatment is very diverse and is expected to overload bacterial repair mechanisms [32]. A synergistic effect could therefore be expected.

The objectives of this study were therefore to analyze the reduction of *Y. enterocolitica* or *B. thermosphacta* inoculated on pork after UV-C and PAA treatment, packaged in modified atmosphere packages (MAP) and stored under cooling conditions for up to 14 days. Besides microbiological analyses, meat quality (e.g., color), and chemical and sensory parameters were investigated.

## 2. Materials and Methods

### 2.1. Culturing of Bacteria

*Y. enterocolitica* field strain M 52 + 2, isolated from the tonsils of a wild boar (Institute for Food Quality and Food Safety, University of Veterinary Medicine Hannover, Foundation, Hannover, Germany) and *B. thermosphacta* DSM 20171 (German Collection of Microorganisms and Cell Cultures, DSMZ, Braunschweig, Germany), isolated from fresh pork sausage, were used for the investigations. The *Y. enterocolitica* field isolate was used in previous investigations [33]. It showed a higher resistance to UV-C radiation than other strains tested, but there were no changes in sensitivity to PAA. Therefore, the field isolate was chosen to investigate the actual effectiveness of the treatment under practical conditions. The isolates were stored at −80 °C in cryotubes until use and were then streaked on Columbia agar plates with sheep blood (Oxoid GmbH, Wesel, Germany). Single colonies were transferred into brain heart infusion (BHI) broth and incubated for 24 h at 30 °C (*Y. enterocolitica*) and 25 °C (*B. thermosphacta*) before suspensions were used for the experiments.

### 2.2. Equipment and Treatment Conditions

For the UV-C irradiation, a UV-Cabinet-HNXE/5 (Light Progress S.r.l., Anghiari, Italy) was used. The cabinet was equipped with five low pressure mercury UV lamps—every lamp with a wavelength emission of 254.7 nm and 40 W. About 10 min before use, the lamps were started in order to ensure sufficient and constant UV intensity (6.8 mW/cm^2^). The intensity was determined with an UV-Sensor SI 1 and the Handheld HI 1 (UV-Technik Meyer GmbH, Ortenberg, Germany) before each trial. To reach a dose of 2040 mJ/cm^2^, samples were UV-treated for 5 min at a distance of 10 cm to the lamps. This dose proved to be effective in preliminary analyses (data not published). To ensure that the samples did not exceed room temperature (about 22 °C), the surface temperature of the meat was measured at three areas per sample directly after UV-irradiation. Therefore, a food infrared thermometer (BP 5F Food-Thermometer, Trotec, Aachen, Germany) was used.

Immediately before each PAA treatment, a PAA solution (Grüssing GmbH, Filsum, Germany) of 2000 ppm was dissolved by mixing 5% PAA solution with high-purity water. This concentration was used, as previous investigations showed that lower PAA concentrations did not significantly reduce the content of these two bacteria on pork (data not shown). In addition, the use of the maximum recommended concentration should show how effectively it works and whether it affects the physicochemical properties. For the spray treatment, a manual spray gun with a 0.5 mm stainless steel nozzle (Universal Spritzpistole Modell W1, Alfred Schütze Apparatebau GmbH, Weyhe-Dreye, Germany) was used. The meat samples were sprayed from a distance of approximately 15 cm with a pressure of 1.5 bar in small circular movements for 30 s with a total of 3 mL ± 0.5 mL PAA or the same volume of sterile high-purity water as control solution.

To determine the most effective treatment conditions for the experiments, two different spray periods (30 s, 60 s), two different PAA concentrations (1200 ppm, 2000 ppm), and four different UV-C doses (408 mJ/cm^2^, 2040 mJ/cm^2^, 4080 mJ/cm^2^, 6120 mJ/cm^2^) were compared in preliminary tests (data not shown).

### 2.3. Material

A total of three replications were carried out. In every replication, the back region of three different female pigs, which were slaughtered at a local slaughterhouse, were used. The meat was transported under cooling conditions to the institute. Twenty four hours after slaughter (24 h postmortem (p.m.)), the *M. longissimus thoracis et lumborum (LM)* of the left and right side was cut from the backbone, and fat and tendons were removed. A 10 g-sample of each *LM* was taken for microbiological analyses to elucidate the general contamination after slaughter, transport, and cutting. For analyses of the overall quality of the meat prior to the specific UV and PAA experiments, four samples—two of each *LM*—were removed between the 13th and 14th vertebrae. One sample per side was used for determination of the meat quality parameters pH value, color, and electric conductivity, and one sample of each side with a thickness of 2.5 cm to assess drip loss, cooking loss, and shear force.

For the specific experiments, in each replication, at 24 h p.m., 18 meat slices of at least 35 g were cut from the meat. They were treated and stored as described below. After treatment and storage, 10 g samples were used for microbiological analysis, as described below, to examine whether the various treatment options had an effect on the microbial content that might already have attached during slaughter or handling of the meat. Further parts of the samples were either used for direct analysis of pH values and color, or analysis of the antioxidant capacity and Mb redox form percentages, as described below.

For the specific inoculation experiments, in each replication, at 24 h p.m., 18 meat slices were cut into round samples with an area of 12.56 cm^2^ using a sterile knife. The samples were inoculated with 40 µL BHI suspension containing either *Y. enterocolitica* or *B. thermosphacta* (7 × 10^7^–4 × 10^8^ cfu/mL) to get a bacteria concentration on meat of about 10^6^ cfu/cm^2^. The bacteria suspensions were evenly spread with an L-shaped spreader (VWR International, Darmstadt, Germany) and placed into a refrigerator for about 20 min before treatment. These inoculated samples were treated and stored as described below. After treatment and storage, the microbiological analyses were performed, as described below. Additionally, for investigation of the effect of a lower amount of bacteria in the inoculum solution, the samples with an area of 12.56 cm^2^ were inoculated with *B. thermosphacta* (1 × 10^6^ cfu/mL) and *Y. enterocolitica* (6 × 10^6^ cfu/mL) to reach a bacterial count of about 10^4^ cfu/cm^2^. The samples were treated in the same way, as described below. Microbiological analyses were only performed on day 1 without packaging.

In three replications, at 24 h p.m., 18 meat slices were cut with a sterile knife into samples with a size of 8 × 8 cm^2^. Each sample was inoculated with 170 µL BHI broth containing either *Y. enterocolitica* or *B. thermosphacta*, as described above. These samples were treated and stored, as described below. Sensory investigation was performed, as described below.

### 2.4. Treatment of Pork

The samples were irradiated with UV-C light and then spray treated with PAA solution. Samples without any treatment, samples treated only with UV-C light, as well as samples sprayed only with PAA or with sterile water instead of PAA and additionally samples with UV-C/water treatment served as controls. Samples were also treated with UV-C light alone or with PAA to measure the effect of individual treatments.

### 2.5. Storage

Each meat sample was transferred separately into a flat polypropylen tray (ES-Plastic GmbH, Hutthurm, Germany), (MultivacT100, Sepp Haggenmueller GmbH& Co. KG, Wolfertschwerden, Germany), packaged in MAP (70% O_2_ and 30% CO_2_) and sealed with a polyethylen-ethylene vinyl alcohol-PP transparent film (Südpack, Ochsenhausen, Germany) in a packing machine. The packaged samples were stored in a refrigerator at 7 °C until further analyses, as the regulation (EC) No. 853/2004 indicates that food business operators must ensure a storage temperature for pig meat of maximum 7 °C. The samples were examined on day 1 (treatment day), day 7 and 14. From the samples for chemical analyses (which were treated but not inoculated), ten grams of meat were taken aseptically from every sample for further microbiological examinations. Furthermore, color and pH were measured, and then the samples were cut into small cubes, frozen in liquid nitrogen, and stored at −80 °C until analyses of antioxidant activity or myoglobin (Mb) redox form percentages. The inoculated round slices were analyzed for their sensory properties as well as their color and swabbed for microbiological examination.

### 2.6. Microbiological Parameters

Ten gram pieces of the samples were put into bags (Stomacher 400 Strainer Bags, Seward limited, Worthing, UK) and diluted 1:10 with sterile saline solution with peptone (0.85% NaCl, 0.1% peptone). The solution was homogenized in a Stomacher (Stomacher 400 Circulator, Seward, Alaska) at 230 rpm for 2 min. Afterwards, serial 10-fold dilutions were prepared. For analysis of total viable counts (TVC), 1 mL of each dilution step was pipetted into a petri dish and filled with 12 to 15 mL plate count agar (CM0325, Oxoid) and was incubated at 30 °C for 72 h. In parallel, 0.1 mL of each dilution step was spread onto SIN-agar (Streptomycin-Inosit-Neutral red-agar) for determination of *Brochothrix* spp. or on CIN-agar (Cefsulodin Irgasan Novobiocin agar, Oxoid, Wesel, Germany) for *Yersinia* spp. analyses and was incubated for 48 h at 25 °C or for 24 h at 30 °C, respectively. For the quantification of *Enterobacteriaceae*, 1 mL of each dilution step was pipetted into a petri dish, filled with 12–15 mL VRBG agar (Violet Red Bile Glucose agar, Oxoid), and incubated for 24 h at 37 °C, according to ISO 21528:2017.

To evaluate the bacterial counts of the previously inoculated round meat samples, the entire surface was swabbed horizontally and vertically for 15 times in a 45° angle to the sample. For this purpose, a sterile applicator (Rotilabo^®^—cotton buds, PP, sterile, small, tip-ø 4–5.5 mm, Paul Boettger GmbH &Co. KG, 94,249 Bodenmais, Germany) was soaked in 1 mL saline peptone solution (0.85% NaCl, 0.1% peptone) and excess liquid was pressed out, so that it was humid, but not wet. After swabbing the sample, the applicator tip was transferred to the saline peptone solution, cut off with a sterile scissor, and vortexed at the highest level for about 5 s. Afterwards, a second swabbing of the sample in the same procedure was performed with a dry applicator and vortexed again for about 5 s. Subsequently, serial 10-fold dilutions of the saline peptone solutions were prepared. The quantification of *Y. enterocolitica* and *B. thermosphacta* was performed as previously described by Reichel et al. [11]. The serial dilutions were plated out and incubated as described above, and then the colony forming units were counted. The detection limits for *B. thermosphacta* and *Y. enterocolitica* were 2.0 log_10_ cfu/cm^2^ and for TVC and *Enterobacteriaceae* 1.0 log_10_ cfu/cm^2^. If no colonies were detected on the respective agar, the half detection limit values (0.7 log_10_ cfu/cm^2^, 1.7 log_10_ cfu/cm^2^) were considered for further statistical analyses.

### 2.7. Sensory Analyses

Sensory analyses were carried out according to Bertram et al. [27]. In three replications, on day 1, 7, and 14, a panel of three experienced persons assessed the appearance and the odor of a sample of each treatment group, as well as the untreated control immediately after opening the tray. Five points could be given overall for each sample and every category: Five points was best (no deviation, typically for fresh meat, no complaints), and one point was worst (unsatisfactory and unacceptable). For the total sensory result, the points for visual aspects were multiplied with 3, added to the points given for odor, and the result was then divided by four. The visual score counted three times, because the meat was packed and the consumers’ purchase decision only depends on color [34]. However, the odor, which is only noticed when the meat is unpacked, had a smaller influence on the overall rating. It might only influence the rebuy decision.

### 2.8. Meat Quality Parameters

The non-inoculated samples were used for meat quality characterization. They were treated and handled in exactly the same way (except for the untreated control) as the inoculated ones.

For measuring the pH values, a portable pH meter (Knick Portamess, Knick GmbH, Berlin, Germany) equipped with a glass electrode (InLab 427, Mettler-Toledo, Urdorf, Switzerland) and a temperature sensor was used. The pH meter was calibrated with two pH standard solutions (pH 7.0 and pH 4.0, Sigma Aldrich Chemie GmbH, Taufkirchen, Germany) before each experiment or in-between, if necessary. The electrode and sensor was centrally inserted into the meat. A total of three measurements per sample were carried out, and the average was calculated.

Color values were measured immediately after unpacking. Lightness (L *), redness (a *), and yellowness (b *) were determined with a colorimeter (Minolta CR 400, Konica-Minolta GmbH, Langenhagen, Germany) 8 mm measuring field. A standard observer of 2° was considered. Before the color analysis, the colorimeter was calibrated using a standard white plate (Konica-Minolta GmbH; Y = 84.0; x = 0.3226; y = 0.3392). Every sample was measured five times, and the mean value was taken for further statistical analyses.

The electrical conductivity (EC, mS/cm) of the untreated, non-inoculated samples was measured three times per sample with an EC-meter (Matthäus GmbH & Co. KG, Nobitz, Germany) for meat characterization. Before each trial, the EC-meter was calibrated by using a specific calibration block (10 mS/cm). The sensors were inserted orthogonal to the meat fibers direction into the center of the samples.

For the drip loss analysis (in %), a sample of about 3 cm was taken from every muscle and weighed 24, 48, and 72 h postmortem. For this, the samples were hung into a plastic box, which was closed and stored at 7 °C until the weight was determined. These samples were also taken for the examination of cooking loss (in %) and the Warner Bratzler shear force test.

For analysis of the cooking loss, the meat samples were weighed directly before cooking, vacuum packed and placed into a laboratory water bath with a temperature of 80 °C. The samples were heated until a core temperature of 75 °C was reached. The samples were cooled to room temperature (about 22 °C), unpackaged and weighed again. The percental difference in weight prior and after cooking was calculated and defined as cooking loss.

In a next step, the Warner–Bratzler shear force test followed. For this purpose, five blocks of 3 × 1 × 1 cm^3^ were cut from each meat sample parallel to meat fiber direction. Each block was positioned in a texture analyzer (Texture Analyzer TA.XT.plus, Stable Micro Systems, Survey, UK) so that the blade was in perpendicular position to the meat fiber direction and each piece of meat was sheared. The blade had a thickness of 1.016 mm. Each value is the average of a total of five measurements per sample.

### 2.9. Chemical Parameters

To determine the antioxidant capacity (AC), an ABTS radical cation solution was prepared according to Re et al. [35]. Distilled water and 2,2′-azinobis-(3-ethyl-benzothiazoline-6-sulfonic acid) diammonium salt (ABTS) were mixed to a final concentration of 7 mM. This solution was mixed with potassium persulfate (K_2_S_2_O_8_, final concentration: 2.45 mM). To ensure a stable oxidation of the ABTS to the ABTS^+^• and a stable absorbance, the ABTS- K_2_S_2_O_8_ mixture was stored in the dark for 12 to 16 h at room temperature. Sample preparations and measurements were performed as described by Sacchetti et al. [36] with some modifications. About 1 g of the frozen meat sample was homogenized on ice with 6 mL distilled water with a Polytron PT 2500 homogenizer for 1 min at 30,000 rpm to extract the hydrophilic fraction. The tube with the homogenized sample was coated with aluminum foil and shaken at 4 °C for 1 h. The homogenate was centrifuged at 2340× *g* (Hermle Z383 K, Hermle Labortechnik, Wehingen, Germany) for 15 min. A mixture was made by adding 3 mL of the ABTS^+^• radical solution to 20 μL of the supernatant or distilled water (control). Before measuring, the ABTS^+^• radical solution was adjusted at 30 °C with water to an extinction of 0.70 ± 0.02 at 734 nm. The sample was measured spectrophotometrically at 734 nm (Evolution 201-UV-VISSpectrophotometer, Thermo Scientific, Langenselbold, Germany), and the absorption after 7 min was used for calculating the AC in µmol Trolox equivalent/g sample. Therefore, 20 µL of 0, 2.5, 5, 7.5, 10, and 15 µM-Trolox standard solutions were added to 3 mL of ABTS^+^• radical solution and analyzed, as described above, to create a calibration curve. For further calculations, only linear calibration curves with a correlation coefficient of at least 0.99 were considered.

The percentages of the Mb redox forms were analyzed according to Kernberger-Fischer et al. [37] with slight modifications. A quantity of 3 g of the nitrogen frozen meat samples was added to 7 mL phosphate-buffered saline (pH 7.4) and homogenized on ice for 1 min at 30,000 rpm (Polytron PT 2500 homogenizer, Kinematica GmbH, Luzern, Switzerland), followed by centrifugation at 35,000× *g* and 4 °C for 30 min (Sorvall RC 5 C Plus, Thermo Scientific). For measuring the amounts of oxymyoglobin (OxyMb), deoxymyoglobin (DeoMb), and metmyoglobin (MetMb), the supernatant was transferred to three semi-micro cuvettes and measured with a spectrophotometer (Evolution 201-UV–VIS-Spectrophotometer, Thermo Scientific) at 503, 525, 557, and 582 nm. The calculation of the Mb redox form amounts were conducted with the equations modified by Tang, Faustman, and Hoagland [38].

### 2.10. Photoreactivation

To investigate possible effects of photoreactivation, overnight cultures of *Y. enterocolitica* and *B. thermosphacta* were prepared on blood agar plates, as described above. Pork from three different batches was purchased from a local supermarket, and slices of 25 g were inoculated with 500 µL bacterial suspension and put into the refrigerator for 20 min. Three samples each were UV-irradiated with a dose of 408 and 2040 mJ/cm^2^ and three control samples were not UV-C irradiated. Of each treatment group, one sample was stored on ice for one hour in the dark, one sample on ice in an enlightened room and one sample was directly homogenized and examined, as described above. Experiments were carried out in triplicate.

### 2.11. Inoculation of a Reduced Bacterial Count

As high bacterial counts of 10^8^ cfu/cm^2^ are generally not present on meat, some of the experiments were carried out with lower concentrations of *B. thermosphacta* (1 × 10^6^ cfu/mL) and *Y. enterocolitica* (6 × 10^6^ cfu/mL) to get an amount of about 1–9 × 10^4^ cfu/cm^2^. The samples were treated in the same way as the others, but were swabbed directly (on day 1) without packaging. These experiments were also run in three independent replications.

### 2.12. Statistical Analysis

All data were statistically analyzed with SAS Enterprise Guide 7.1 (SAS Institute Inc., Cary, NC, USA) considering the following model:

Y_ij_ = µ + T_i_ + R_j_ + ε_jj_

where Y_ij_ = observation value; µ = overall mean, D_i_ = fixed effect of treatment (Control, UV-C, UV-C + H_2_O, H_2_O, PAA, UV-C + PAA); R_j_ = random effect of replication; ε_ij_ = random error.

To compare the different treatment groups, the TUKEY multiple comparison test was used. If the *p*-value was 0.05 and lower, the result was considered significant (*p* ≤ 0.05). All experiments were performed three times.

## 3. Results and Discussion

For the presentation of the results and their discussion, the following must be taken into account: For the UV-C-treated samples, the untreated samples served as controls, while for the PAA treatment, the water-treated samples were used as controls (to record the rinsing effect).

For the presentation of the effect of the combination of UV-C and PAA, the single-treated samples as well as the UV-C/water-combination were used as controls.

### 3.1. Physicochemical and Microbial Quality of Pork Before Treatment

Meat quality parameters of the pork were measured 24 h p.m. to assess the initial parameters and to ensure that there were no quality deviations that could distort the results of the physicochemical analyses (Table 1). The meat quality results mainly agree with the results presented by Reichel et al. [11], Ruusunen et al. [39], Werner et al. [40], Mörlein et al. [41], and Kim et al. [42]. Differences are due to different endogenic (i.e., sex, genetic) or exogenic (i.e., transport, slaughter, analytical methods) factors. The TVC, *Enterobacteriaceae*, *Yersinia* spp., and *Brochothrix* spp. results, which are dependent on the health status of the pig as well as the hygienic conditions at the slaughterhouse and during storage and distribution [7], indicate that the animals were slaughtered at good hygienic conditions. All in all, the used meat represents common pork.

### 3.2. Effects of PAA and UV-C Treatments on the Microbiological Survival on Pork During Storage

The antimicrobial effects of treatments with PAA, UV-C, and their combination on pork inoculated with *Y. enterocolitica* or *B. thermosphacta* are presented in Table 2. Considering the single treatments, on storage days 1 and 7 UV-C treatment resulted in a significant reduction of both bacterial species in comparison to the untreated (control) samples, whereas PAA treatment significantly reduced only *B. thermosphacta* on days 1 and 7 of storage compared to the H_2_O treated samples. After combined UV-C/PAA treatment compared to the UV-C/water treatment (where the PAA treatment has been replaced by a water treatment), a significant reduction of bacteria was only found for *Y. enterocolitica* inoculated pork on storage day 14.

The effect of UV-C irradiation on *B. thermosphacta* and *Y. enterocolitica* indicates that UV-C treatment can effectively reduce bacterial counts. These results mainly agree with Reichel et al. [11], who presented a significant reduction of both *Y. enterocolitica* and *B. thermosphacta* on pork on storage days 1, 7, 14, irradiated with UV-C doses of 408 mJ/cm^2^ and 2040 mJ/cm^2^. Isohanni et al. [31] who treated broiler meat and skin with UV-C doses of 9.4, 18.8, and 32.9 mJ/cm^2^ presented significant reductions of *Campylobacter* (*C.) jejuni*. Moreover, Haughton et al. [30] found significantly lower *C. jejuni*, *S.* Enteritidis and *E. coli* numbers on chicken meat after treatments with UV-C doses up to 192 mJ/cm^2^. Reichel et al. [11] presented in in vitro studies higher reductions of up to 4.0 log_10_ cfu/mL of *Y. enterocolitica* and *B. thermosphacta* at lower UV-C doses of up to 30 mJ/cm^2^. However, there are several reasons for the low reduction of the bacteria on pork and the high variation of the results compared to the in vitro studies. One is that UV light only reacts on surfaces not penetrating the matrix [43] and bacteria might shield each other from the UV-C rays [9,44,45]. Another reason might be the rough surface of the meat with pores and caverns, which protect the bacteria from the UV-C light and complicate the recovery of the bacteria. Furthermore, the meat proteins may lead to absorption of the UV-C rays [11,45,46]. Low reduction results might also be caused by reparation of the UV-C generated DNA damages either in the light, catalyzed by the photolyase, or in the dark by several enzymes that repair DNA damages by excision [47,48]. To clarify the latter assumption, we analyzed how *B. thermosphacta* and *Y. enterocolitica* inoculated on pork, treated with doses of 408 and 2040 mJ/cm^2^, and stored for 60 min in the dark or light, grew in comparison to samples analyzed directly after UV-C treatment. Growth would indicate repair of the DNA damages after UV-C treatment. However, since UV-C treated samples had significantly lower bacterial levels directly after treatment and after 60 min of light or dark storage compared to the untreated control samples, it could be suggested that photoreactivation does not effectively influence the bacterial counts (Table 3).

Reichel et al. [49] also found no photoreactivation effects of *B. thermosphacta* and *Y. enterocolitica*, which were inoculated on ham, treated with UV-C doses of 408 and 4080 mJ/cm^2^, and stored in the dark and light for 60 min. Reichel et al. [11] presented through in vitro studies that after 60 min of light storage, higher bacterial counts were determined than directly after treatment of both bacterial species with increasing UV-C doses up to 30 mJ/cm^2^. These data indicate that although no photoreactivation of the bacteria was seen on pork, the storage period of 60 min was sufficient to evaluate this repair mechanism. It can be suggested that high UV-C doses, necessary for a significant treatment effect on meat, generate bigger DNA damages, resulting in reduction/overburden of the different DNA repair systems—during light repair, the photolyase enzyme system, and during dark repair, several enzymes that repair DNA damages by excision of dimers [47,48].

The presented effects of PAA on *B. thermosphacta* on days 1 and 7 of storage indicate that PAA spray treatment can extend the shelf life of pork. This result agrees basically with the study of Bertram et al. [27,50], who applied 1200 ppm PAA by spraying turkey, chicken breast, and drumsticks with skin. They showed a significant decrease in *C. jejuni* counts and TVC on days 6 and 12 of storage. Smith et al. [51] found significant reductions of *C. jejuni* on broiler carcasses after treatments with 100 and 200 ppm PAA by immersion or spraying. Nagel et al. [29] presented significantly lower *S.* Typhimurium and *C. jejuni* counts on inoculated poultry carcasses after treatments with 400 ppm or 1000 ppm PAA in a post-chill immersion tank, whereas Ellebracht et al. [28] or Penney et al. [52] treated beef with 200 ppm or 180 ppm PAA, respectively, resulting in significant reductions of *E. coli* and *S.* Typhimurium counts (only Ellebracht et al. [28]). In similar experiments, Cap et al. [53] presented significant reductions of Shiga toxin-producing *E. coli*. The different study conditions, the varying susceptibilities of the used bacteria, and particularly the meat matrix may have caused the different effects also within the present study. The assumption that differences in the susceptibility of bacteria play a role is also supported by Aarnisalo et al. [54], Poimenidou et al. [55], or Skowron et al. [56]. They analyzed the minimal inhibitory concentrations (MICs) of 6, 12 or 6 *Listeria* (*L.*) *monocytogenes* strains, respectively, against PAA or PAA containing disinfectants, and detected differences in the MIC values. Similar to this, Bertram et al. [27] analyzed the MICs of 25 *C. jejuni* and *C. coli* isolates, and also found some variation in the values. Considering the effect of the meat matrix, Bertram et al. [27] clearly showed that growth of *C. jejuni* is inhibited at concentrations of 2 to 8 ppm PAA in in vitro (MIC) experiments, while 1200 ppm PAA is necessary to reduce the bacterial species on turkey and broiler skin by approximately 1.0 log_10_ cfu/g skin.

The UV-C/PAA results indicate that after a significant reduction of bacteria due to UV-C application or PAA spraying, further treatment with another preservation method did not improve the treatment effects. However, studies on a combination of these two preservation methods on pork initially inoculated with *B. thermosphacta* or *Y. enterocolitica* have not been published yet. There are in vitro studies that have shown a reduction of the amount of *L. monocytogenes* using disinfectants containing chemicals such as hydrogen peroxide or phosphonic acid in addition to PAA. However, these studies were not carried out on meat products [54,56]. Other studies found reductions in *Salmonella* or *E. coli* counts after treatment of chicken carcasses with chemicals such as lauryl ethyl arginate (LAE), acidic calcium sulfate, polylysine, or vinegar solution in combinations [57,58]. Sukumaran et al. [59] treated chicken skins with PAA and bacteriophages and found beneficial effects on *Salmonella* levels. The absence of significant reductions was probably due to the meat matrix, which partly also influenced the effectiveness of individual treatments with UV-C rays or PAA.

To avoid that possible treatment-related reductions in bacterial counts reach the detection limit of 2.0 log_10_ cfu/cm^2^ and thus could not be detected, the samples were inoculated with high bacterial counts at the beginning of the study. As contamination of meat is often lower, especially in slaughterhouses or meat processing plants that comply with hygiene standards, lower bacterial counts of 1–6 × 10^6^ cfu/mL were also inoculated in further experiments. Using the lower inoculum concentration, both bacterial species showed a significant reduction in the bacterial counts after UV-C treatment compared to their control. In contrast, the PAA treatment did not result in a significant reduction of both species when using the lower inoculum concentration. After combined UV-C/PAA treatments, the bacterial counts were similar to those after UV-C/water treatment, but compared to the PAA treatment alone the *Brochothrix* and *Yersinia* counts were significantly reduced when using a lower inoculum concentration (Table 4).

The water treatment caused significant reductions of *B. thermosphacta* up to 0.87 log_10_ cfu/cm^2^ (day 1) and of *Y. enterocolitica* up to 0.98 log_10_ cfu/cm^2^ (day 1) compared to the untreated samples. This effect was also shown by Bertram et al. [27], who treated chicken drumsticks with a water spray with the same distance and water volume and achieved reductions for *C. jejuni* of 0.77 log_10_ cfu/g (day 1), 0.64 log_10_ cfu/g (day 6), and 0.57 log_10_ cfu/g (day 12). Nagel et al. [29], who treated chicken carcasses 20 s in a water dip (in 1.5 L) achieved a more than 0.5 log cfu/sample reduction of *S.* Typhimurium und *C. jejuni*. In the present study, we considered the water treatment as control in relation to the PAA or PAA/UV treatment assuming that washing down effects, as presented also by the other studies, might be detectable due to insufficient attachment of the bacteria to the meat surface.

The results after UV-C treatments were similar at both low and high inoculum concentrations. However, the missing effect of PAA on *B. thermosphacta* and the significantly lower results after combined UV-C/PAA treatments compared to the PAA results are in contrast to the findings with the higher inoculum concentrations. The data with low inoculum concentrations rather show that UV-C irradiation is more effective than PAA.

That the initial reduction in bacterial counts did not increase again during further storage is probably caused by the low temperature and the inhibitory effect of the oxygen within the modified atmosphere as shown by Zhang et al. [60], and was also demonstrated for the control samples within the present study.

### 3.3. Effects of PAA, UV-C Treatment, and the Combination UV-C/PAA on the Color, pH, Mb Redox form Percentages and AC Results of Inoculated Meat

The meat color is an important factor influencing the purchase behavior of the consumers, especially, if the meat is packed. This is because other sensory parameters like smell, taste, or texture cannot be assessed at retail level. A bright red color is an indicator for freshness and is influenced by the Mb content and the percentages of the Mb redox forms [61]. With regard to the effects of the UV-C and PAA treatments alone and in combination on the color results, no significant effects could be found (Table 5 and Table 6).

The present results agree with those presented by Reichel et al. [11], who also found no significant effects of UV-C treatment on the color of pork with doses of 408 mJ/cm^2^ and 2040 mJ/cm^2^ and subsequent MAP storage for 14 days. This is in accordance with Lyon et al. [62], who found no significant color changes of chicken breasts after UV-C irradiation with a dose of 300 mJ/cm^2^ and after a storage period of 7 days. In contrast to the present study, Stermer et al. [15] presented significantly higher a* values of beef after UV-C irradiation with doses of 500 mJ/cm^2^. Wallner-Pendleton et al. [63] found significantly lower L* values after UV-C irradiation of broiler legs with a dose of 82.56 mJ/cm^2^ at day 0 and 10 of storage. However, color values of the chicken breast were not influenced by the treatments. Park et al. [16] also found significantly lower L* values of chicken breast meat irradiated with UV-C doses of 60–3600 mJ/cm^2^, and significantly higher a* values of chicken breast meat irradiated with UV-C doses of 1800–3600 mJ/cm^2^.

With regard to the PAA results, Bertram et al. [27,50] also found no effects of PAA on the color of turkey and broiler breast meat sprayed with 1200 ppm PAA solution. In contrast to this, ground beef patties showed significantly higher L* values if treated with 200 ppm PAA compared to the untreated samples (Quilo et al. [25]). In a similar study, Quilo et al. [26] found on days 0, 1, 2, 3, and 7 significantly higher a * results of the ground beef after treatments with 200 ppm PAA.

The missing effects of the combination of UV-C and PAA are comprehensible since no effects of color by using the single applications were achieved in the presented study either. Unfortunately, no comparable studies have been published. However, despite the partly contradictory results of the UV-C and PAA treatments on the color results in other studies, the present study indicates that both preservations methods alone or in combination could be used without negative impact on the color of meat.

With regard to the Mb redox form percentages, no significant effects of the UV-C and PAA alone and in combination was obtained (Table 7).

Reichel et al. [11] also found no impact of the UV-C treatment on the Mb redox form percentages of pork, whereas Bertram et al. [27,50] found no significant effect of the PAA treatment on the Mb parameters of broiler and turkey breast muscle. No other studies have been published which analyzed the Mb redox forms after combined UV-C and PAA treatments. However, the Mb results seem to be comprehensible if one considers the almost similar color results. During storage, OxyMb is oxidized to MetMb, which causes a color change from red to brown and may negatively affect the consumers’ behavior [64].

No significant effects of UV-C and PAA alone or in combination on the pH values as well as AC results could be found in the present study (Table 8).

The pH results agree with those of Park et al. [16] or Reichel et al. [11], who also found no effects of UV-C treatment on this parameter. No influence of a PAA treatment on the pH results was also seen by Bertram et al. [27,50] or Quilo et al. [25]. In addition, Reichel et al. [11] also found no significant effects on the AC after UV-C irradiation with 408 and 2040 mJ/cm^2^ compared to the untreated pork samples for all days of storage (1, 7, 14). In contrast, Bertram et al. [50] presented similar AC values of broiler meat, when treated with 1200 ppm PAA or water, the solvent of the PAA. The AC value determination quantifies the concentration of antioxidant substances like tocopherol, ascorbic acid, or glutathione within the tissue. As these substances might reduce the oxidation of molecules such as proteins and lipids and thus prevent oxidative stress within the cells [50,65], a reduction in AC results indicates an oxidative influence on the tissue. This can result in negative effects on the physicochemical properties of the meat such as color, Mb redox forms, or lipid peroxidation: The latter might also affect human health [66]. However, as in the present study, neither UV-C nor PAA changed the AC values, an oxidative effect of these preservation methods could be excluded, even considering the color or Mb results presented. Therefore, it is comprehensible that the combined treatment with UV-C and PAA also had no impact on the AC as well as color or Mb results.

### 3.4. Effects of PAA, UV-C Treatment and the Combination UV-C/PAA on Sensory Effects of the Pork

The results of the sensory analyses are shown in Table 9.

UV-C treatment caused a significant decrease in the sensory results on day 1 of storage compared to the untreated samples. On day 1, pork, inoculated with *Yersinia* and treated with PAA, was rated lower in sensory parameters. This effect of the PAA was also found on day 7, if the pork was inoculated with *Brochothrix.* Samples which were treated with the combination of UV-C/PAA had significantly lower sensory results on day 7 when inoculated with *Brochothrix* and on day 14 when inoculated with both bacterial species compared to the UV-C water combination. The sensory results are in agreement with results presented by McLeod et al. [46], who found odor changes of chicken fillets after UV-C irradiation with a dose of 3000 mJ/cm^2^, which is a relatively high dose. Park et al. [16], who irradiated chicken breasts with higher UV-C doses up to 3600 mJ/cm^2^, also found significant decreases on the sensory results (color, texture, flavor, appearance, overall acceptability), which were dependent on the UV-C dose (the higher the dose the worse the overall acceptability). Studies that applied lower UV-C doses reported no significant differences of the sensory quality after UV-C irradiation with doses of 32.9 mJ/cm^2^ (chicken) and up to 500 mJ/cm^2^ (beef) [15,31]. Bertram et al. [50] found no significant differences of the sensory results after treating chicken fillets with 1200 ppm PAA, despite a slightly acetic acid odor on day 1 as well as on days 6 and 12 of storage. The sensory alterations after UV treatment might be caused by ozone absorption, nitrogenoxides, and photochemical changes of the meat lipids [8], whereas alteration due to PAA seems to be related to release of acetic acid from the PAA.

## 4. Conclusions

The study shows that in contrast to the single treatments, which showed reductions up to 1.60 log_10_ cfu/cm^2^ for *Y. enterocolitica* and 1.64 log_10_ cfu/cm^2^ for *B. thermosphacta* after UV-C irradiation and up to 1.14 log_10_ cfu/cm^2^ for *Y. enterocolitica* and 1.03 log_10_ cfu/cm^2^ for *B. thermosphacta* after PAA treatment compared to their controls, a combined UV-C/PAA treatment is not effective to reduce *Y. entercolitica* and *B. thermosphacta* counts on pork compared to controls. This is unexpected, as treatment with UV-C and, to a lesser extent, with PAA clearly reduces the number of these bacteria inoculated on pork. Therefore, a combined effect of both preservation methods could not be recommended for this purpose.

## Figures and Tables

**Table 1:** Least square mean (LSM) and standard error (SE) values of the quality parameters of pork taken for the study (*n* = 6).

Parameter		Parameter	
pH 24 h p.m.	5.35 ± 0.04	Cooking loss (%)	26.47 ± 1.09
EC 24 h p.m. ^1^	8.40 ± 0.97	Shear force (N)	29.47 ± 2.65
L* 24 h p.m.	55.22 ± 0.64	Total viable count ^3^	2.30 ± 0.82
a* 24 h p.m.	7.20 ± 0.58	*Enterobacteriaceae* ^3^	0.7 ± 0.0
b* 24 h p.m.	5.38 ± 0.25	*Yersinia* spp. ^3^	1.7 ± 0.0
Drip loss (%) ^2^	6.22 ± 0.51	*Brochothrix* spp. ^3^	1.7 ± 0.0

^1^ EC = electrical conductivity in mS/cm; ^2^ drip loss (calculated from the weight 72 h post mortem (p.m.) subtracted from the weight 24 h p.m.); ^3^ all values in log_10_ colony forming units/g meat. The detection limits were 1.0 log_10_ cfu/g (Total viable count and *Enterobacteriaceae*) or 2.0 log_10_ cfu/g (*Yersinia* spp. and *Brochothrix* spp.); if no bacterial growth was determined, the half detection limit values were considered for further analysis.

**Table 2:** LSM ± SE of *Yersinia enterocolitica* and *Brochothrix thermosphacta* counts on pork previously inoculated with these bacteria after UV-C, peracetic acid (PAA), and H_2_O treatments alone and in combination after 1, 7, and 14 days of storage (*n* = 3).

Treatment	Day 1		Day 7		Day 14	
	*Yersinia* ^1^	*Brochothrix* ^1^	*Yersinia* ^1^	*Brochothrix* ^1^	*Yersinia* ^1^	*Brochothrix* ^1^
Control	6.34 ^a^ ± 0.19	5.76 ^a^ ± 0.12	6.54 ^a^ ± 0.62	4.80 ^a^ ± 0.16	6.24 ^a^ ± 0.18	4.56 ^a^ ± 0.44
UV-C ^2^	4.98 ^b^ ± 0.12	4.62 ^bc^ ± 0.14	4.94 ^b^ ± 0.32	3.75 ^bc^ ± 0.35	5.01 ^abc^ ± 0.47	2.92 ^ab^ ± 0.42
H_2_O ^3^	5.36 ^b^ ± 0.22	4.89 ^b^ ± 0.29	5.75 ^ab^ ± 0.43	4.30 ^ab^ ± 0.04	5.78 ^ab^ ± 0.43	3.41 ^ab^ ± 0.09
PAA ^4^	5.70 ^ab^ ± 0.30	4.28 ^cd^ ±0.21	4.90 ^b^ ± 0.31	3.31 ^cd^ ± 0.06	4.64 ^bc^ ± 0.17	2.38 ^b^ ± 0.38
UV-C/H_2_O ^5^	5.04 ^b^ ± 0.24	4.24 ^cd^ ± 0.31	5.00 ^b^ ± 0.65	3.52 ^cd^ ± 0.13	5.61 ^ab^ ± 0.31	2.90 ^b^ ± 0.43
UV-C/PAA ^6^	5.16 ^b^ ± 0.03	4.00 ^d^ ± 0.30	4.82 ^b^ ± 0.22	2.99 ^d^ ± 0.11	4.08 ^c^ ± 0.35	2.19 ^b^ ± 0.35

^1^ All values in log_10_ colony forming units/cm^2^ meat the detection limits were 2.0 log_10_ cfu/cm^2^; if no bacterial growth was determined the half detection limit values were considered for further analysis; ^2^ 2040 mJ/cm^2^; ^3^ 30 s water spraying; ^4^ 30 s 2000 ppm PAA spraying; ^5^ 2040 mJ/cm^2^ and 30 s water spraying; ^6^ 2040 mJ/cm^2^ and 30 s 2000 ppm PAA spraying; ^abcd^ mean values in a column with a different letter differ significantly (*p* ≤ 0.05) by Tukey’s test.

**Table 3:** LSM ± SE of *Yersinia enterocolitica* and *Brochothrix thermosphacta* counts on pork previously inoculated with these bacteria directly after treatments and after one hour of storage either in the light or in the dark to elucidate the effect of photoreactivation (*n* = 3).

Doses	Direct		Light		Dark	
	*Yersinia* ^1^	*Brochothrix* ^1^	*Yersinia* ^1^	*Brochothrix* ^1^	*Yersinia* ^1^	*Brochothrix* ^1^
0 mJ/cm^2^	6.66 ^a^ ± 0.05	6.24 ^a^ ± 0.07	6.56 ^a^ ± 0.07	6.05 ± 0.22	6.63 ^a^ ± 0.07	6.17 ^a^ ± 0.09
408 mJ/cm^2^	6.13 ^b^ ± 0.05	5.92 ^b^ ± 0.04	6.15 ^b^ ± 0.06	5.96 ± 0.01	6.13 ^b^ ± 0.00	5.89 ^b^ ± 0.08
2040 mJ/cm^2^	6.06 ^b^ ± 0.06	5.83 ^b^ ± 0.07	5.96 ^b^ ± 0.09	5.85 ± 0.04	5.99 ^b^ ± 0.04	5.87 ^b^ ± 0.02

^1^ All values in log_10_ colony forming units/cm^2^ meat; the detection limits were 2.0 log_10_ cfu/cm^2^; if no bacterial growth was determined, the half detection limit values were considered for further analysis; ^ab^ mean values in a column with a different letter differ significantly (*p* ≤ 0.05) by Tukey’s test.

**Table 4:** LSM ± SE of *Yersinia enterocolitica* and *Brochothrix thermosphacta* counts on pork inoculated with lower concentrations of both bacterial species (*Y. enterocolitica* 6 × 10^6^ cfu/mL inoculum broth; *B. thermosphacta* 1 × 10^6^ cfu/mL inoculum broth) after UV-C, peracetic acid (PAA), and H_2_O treatment alone and in combinations (*n* = 3).

Treatment	Day 1	
	*Yersinia* ^1^	*Brochothrix* ^1^
Control	4.46 ^a^ ± 0.06	3.56 ^a^ ± 0.19
UV-C ^2^	2.94 ^b^ ± 0.16	2.03 ^cd^ ± 0.06
H_2_O ^3^	3.96 ^a^ ± 0.23	3.05 ^ab^ ± 0.23
PAA ^4^	4.00 ^a^ ± 0.30	2.36 ^bc^ ± 0.07
UV-C/H_2_O ^5^	2.39 ^b^ ± 0.10	1.60 ^d^ ± 0.17
UV-C/PAA ^6^	2.74 ^b^ ± 0.17	1.59 ^d^ ± 0.08

^1^ All values in log_10_ colony forming units/cm^2^ meat; the detection limits were 2.0 log_10_ cfu/cm^2^; if no bacterial growth was determined, the half detection limit values were considered for further analysis; ^2^ 2040 mJ/cm^2^; ^3^ 30 s water spraying; ^4^ 30 s 2000 ppm PAA spraying; ^5^ 2040 mJ/cm^2^ and 30 s water spraying; ^6^ 2040 mJ/cm^2^ and 30 s 2000 ppm PAA spraying; ^abcd^ mean values in a column with a different letter differ significantly (*p* ≤ 0.05) by Tukey’s test.

**Table 5:** LSM ± SE of the lightness (L*), redness (a*), and yellowness (b*) values of pork after UV-C, peracetic acid (PAA) and H_2_O treatment alone and in combination, inoculated with *Brochothrix thermosphacta*, after 1, 7, and 14 days of storage (*n* = 3).

Treatment	Day 1			Day 7			Day 14		
	L*	a*	b*	L*	a*	b*	L*	a*	b*
Control	57.8 ^b^ ± 0.5	8.5 ± 1.0	9.4 ± 1.0	61.5 ^ab^ ± 1.5	8.1 ^a^ ± 0.7	10.1 ± 0.8	61.4 ^b^ ± 1.3	6.8 ± 0.4	9.6 ± 1.1
UV-C ^1^	57.8 ^b^ ± 0.5	7.9 ± 0.9	9.5 ± 0.7	60.0 ^b^ ± 1.7	8.1 ^a^ ± 0.3	10.2 ± 0.2	61.3 ^b^ ± 1.0	6.1 ± 0.1	9.9 ± 0.7
H_2_O ^2^	58.7 ^ab^ ± 1.1	8.5 ± 0.7	9.3 ± 0.8	62.2 ^ab^ ± 0.6	7.4 ^ab^ ± 0.6	9.6 ± 0.6	63.5 ^ab^ ± 2.0	6.4 ± 0.6	9.8 ± 0.5
PAA ^3^	60.9 ^a^ ± 1.3	7.0 ± 0.9	9.0 ± 1.1	63.0 ^a^ ± 0.8	6.4 ^b^ ± 0.2	9.9 ± 0.6	65.1 ^a^ ± 1.7	5.7 ± 0.4	10.6 ± 1.0
UV-C/H_2_O ^4^	58.7 ^ab^ ± 1.0	7.8 ± 0.8	9.3 ± 0.8	61.5 ^ab^ ± 1.0	7.7 ^ab^ ± 0.5	10.4 ± 0.6	64.2 ^ab^ ± 0.7	5.8 ± 0.3	9.9 ± 0.7
UV-C/PAA ^5^	59.6 ^ab^ ± 1.1	7.4 ± 0.2	9.6 ± 0.5	63.6 ^a^ ± 1.2	6.5 ^b^ ± 0.2	10.8 ± 0.3	66.4 ^a^ ± 2.3	5.2 ± 0.4	10.8 ± 0.4

^1^ 2040 mJ/cm^2^; ^2^ 30 s water spraying; ^3^ 30 s 2000 ppm PAA spraying; ^4^ 2040 mJ/cm^2^ and 30 s water spraying; ^5^ 2040 mJ/cm^2^ and 30 s 2000 ppm PAA spraying; ^ab^ mean values in a column with a different letter differ significantly (*p* ≤ 0.05) by Tukey’s test.

**Table 6:** LSM ± SE of the lightness (L*), redness (a*), and yellowness (b*) values of pork after UV-C, peracetic acid (PAA) and H_2_O treatment alone and in combinations, inoculated with *Yersinia enterocolitica*, after 1, 7, and 14 days of storage (*n* = 3).

Treatment	Day 1			Day 7			Day 14		
	L*	a*	b*	L*	a*	b*	L*	a*	b*
Control	56.8 ± 0.3	8.1 ± 0.4	9.0 ± 0.4	58.2 ^b^ ± 0.4	8.4 ± 0.6	9.5 ± 0.4	59.5 ^b^ ± 0.8	7.1 ± 0.2	9.0 ± 0.3
UV-C ^1^	58.3 ± 0.5	7.7 ± 0.6	9.3 ± 0.5	59.3 ^ab^ ± 1.5	7.2 ± 0.6	9.0 ± 0.8	60.8 ^ab^ ± 1.0	7.1 ± 0.1	9.9 ± 0.5
H_2_O ^2^	59.4 ± 1.2	8.4 ± 0.7	9.5 ± 0.4	60.9 ^ab^ ± 0.7	8.0 ± 0.6	9.7 ± 0.5	62.5 ^ab^ ± 0.5	6.6 ± 0.3	9.3 ± 0.4
PAA ^3^	58.4 ± 1.4	6.9 ± 0.7	8.5 ± 0.7	61.9 ^a^ ± 0.0	7.2 ± 0.4	9.7 ± 0.4	63.2 ^a^ ± 0.5	6.4 ± 0.4	10.3 ± 0.7
UV-C/H_2_O ^4^	59.3 ± 1.2	7.6 ± 0.9	9.2 ± 0.9	60.6 ^ab^ ± 1.0	7.6 ± 0.4	9.7 ± 0.4	62.4 ^ab^ ± 0.6	6.8 ± 0.5	9.9 ± 0.6
UV-C/PAA ^5^	60.0 ± 0.7	7.0 ± 0.1	9.1 ± 0.4	60.4 ^ab^ ± 0.9	7.1 ± 0.5	10.1 ± 0.9	62.6 ^ab^ ± 0.4	5.9 ± 0.2	10.1 ± 0.4

^1^ 2040 mJ/cm^2^; ^2^ 30 s water spraying; ^3^ 30 s 2000 ppm PAA spraying; ^4^ 2040 mJ/cm^2^ and 30 s water spraying; ^5^ 2040 mJ/cm^2^ and 30 s 2000 ppm PAA spraying; ^ab^ mean values in a column with a different letter differ significantly (*p* ≤ 0.05) by Tukey’s test.

**Table 7:** LSM ± SE of the Oxy-myoglobin (Oxy-Mb), Met-myoglobin (Met-Mb), and Deoxy-Myoglobin (Deoxy-Mb) percentages of pork after UV-C, peracetic acid (PAA), and H_2_O treatments alone and in combination after 1, 7, and 14 days of storage (*n* = 3).

Treatment	Day 1			Day 7			Day 14		
	Oxy-Mb	Met-Mb	Deoxy-Mb	Oxy-Mb	Met-Mb	Deoxy-Mb	Oxy-Mb	Met-Mb	Deoxy-Mb
Control	39.7 ± 3.0	42.7 ± 1.8	17.3 ± 1.4	32.1 ± 2.2	50.6 ± 2.0	17.1 ± 0.2	27.2 ± 3.8	54.7 ± 3.2	17.9 ± 0.8
UV-C ^1^	40.2 ± 2.9	42.6 ± 2.6	16.7 ± 0.0	32.1 ± 2.1	49.8 ± 1.7	17.9 ± 0.9	30.1 ± 0.8	52.1 ± 0.8	17.4 ± 1.0
H_2_O ^2^	43.0 ± 1.1	40.4 ± 1.5	16.2 ± 0.4	31.7 ± 2.1	49.7 ± 2.3	18.3 ± 0.6	27.3 ± 2.8	53.9 ± 2.0	18.6 ± 0.9
PAA ^3^	38.1 ± 2.7	44.2 ± 2.5	17.4 ± 0.5	27.5 ± 0.8	53.7 ± 0.1	18.7 ± 0.8	25.2 ± 1.2	56.5 ± 0.8	18.1 ± 0.6
UV-C/H_2_O ^4^	39.0 ± 4.9	43.2 ± 4.1	17.5 ± 1.2	34.3 ± 1.8	47.9 ± 0.5	17.5 ± 1.5	25.7 ± 3.4	55.6 ± 2.5	18.4 ± 1.1
UV-C/PAA ^5^	38.5 ± 5.3	44.2 ± 4.2	17.0 ± 1.3	26.4 ± 1.5	53.7 ± 1.1	19.9 ± 1.2	26.0 ± 3.6	55.8 ± 2.7	18.0 ± 1.1

^1^ 2040 mJ/cm^2^; ^2^ 30 s water spraying; ^3^ 30 s 2000 ppm PAA spraying; ^4^ 2040 mJ/cm^2^ and 30 s water spraying; ^5^ 2040 mJ/cm^2^ and 30 s 2000 ppm PAA spraying.

**Table 8:** LSM ± SE of the pH values and antioxidant capacity (AC) values of pork after UV-C, peracetic acid (PAA), and H_2_O treatments alone and in combination after 1, 7, and 14 days of storage (*n* = 3).

Treatment	Day 1		Day 7		Day 14	
	pH	AC ^1^	pH	AC ^1^	pH	AC ^1^
Control	5.37 ± 0.03	3.7 ± 0.3	5.41 ± 0.09	4.2 ^ab^ ± 0.2	5.42 ± 0.05	3.1 ± 0.2
UV-C ^2^	5.36 ± 0.04	4.0 ± 0.4	5.43 ± 0.09	4.3 ^a^ ± 0.3	5.48 ± 0.04	3.4 ± 0.2
H_2_O ^3^	5.35 ± 0.03	3.9 ± 0.2	5.46 ± 0.11	3.4 ^bc^ ± 0.3	5.44 ± 0.03	2.9 ± 0.3
PAA ^4^	5.36 ± 0.03	4.0 ± 0.6	5.41 ± 0.03	3.2 ^c^ ± 0.3	5.40 ± 0.03	2.9 ± 0.3
UV-C/H_2_O ^5^	5.33 ± 0.03	3.8 ± 0.3	5.40 ± 0.05	3.6 ^abc^ ± 0.3	5.42 ± 0.04	3.2 ± 0.3
UV-C/PAA ^6^	5.36 ± 0.07	4.0 ± 0.1	5.36 ± 0.04	3.4 ^bc^ ± 0.2	5.42 ± 0.04	2.7 ± 0.1

^1^ All values in µmol Trolox eq./g meat; ^2^ 2040 mJ/cm^2^; ^3^ 30 s water spraying; ^4^ 30 s 2000 ppm PAA spraying; ^5^ 2040 mJ/cm^2^ and 30 s water spraying; ^6^ 2040 mJ/cm^2^ and 30 s 2000 ppm PAA spraying; ^abc^ mean values in a column with a different letter differ significantly (*p* ≤ 0.05) by Tukey’s test.

**Table 9:** Sensory results (LSM ± SE) of pork after UV-C, peracetic acid (PAA), and H_2_O treatments alone and in combination of with *Yersinia enterocolitica* or *Brochothrix thermosphacta* inoculated pork samples after 1, 7, and 14 days of storage (*n* = 3).

Treatment	Day 1		Day 7		Day 14	
	*Yersinia*	*Brochothrix*	*Yersinia*	*Brochothrix*	*Yersinia*	*Brochothrix*
Control	4.9 ^a^ ± 0.1	5.0 ^a^ ± 0.0	3.6 ^a^ ± 0.2	3.9 ^a^ ± 0.0	3.4 ^a^ ± 0.1	3.1 ^a^ ± 0.4
UV-C ^1^	3.8 ^b^ ± 0.3	4.0 ^bc^ ± 0.3	3.1 ^ab^ ± 0.3	3.5 ^a^ ± 0.2	3.2 ^a^ ± 0.2	2.9 ^a^ ± 0.4
H_2_O ^2^	4.6 ^a^ ± 0.1	4.3 ^ab^ ± 0.2	2.7 ^bc^ ± 0.3	3.3 ^a^ ± 0.0	2.8 ^ab^ ± 0.2	2.9 ^a^ ± 0.2
PAA ^3^	3.7 ^b^ ± 0.1	3.8 ^bcd^ ± 0.1	2.3 ^c^ ± 0.3	2.5 ^b^ ± 0.3	2.2 ^bc^ ± 0.2	2.3 ^ab^ ± 0.3
UV-C/H_2_O ^4^	3.7 ^b^ ± 0.2	3.3 ^cd^ ± 0.1	2.7 ^bc^ ± 0.3	3.4 ^a^ ± 0.1	2.8 ^ab^ ± 0.1	2.5 ^a^ ± 0.1
UV-C/PAA ^5^	3.2 ^b^ ± 0.1	3.1 ^d^ ± 0.1	2.1 ^c^ ± 0.3	2.1 ^b^ ± 0.0	1.7 ^c^ ± 0.3	1.5 ^b^ ± 0.3

^1^ 2040 mJ/cm^2^; ^2^ 30 s water spraying; ^3^ 30 s 2000 ppm PAA spraying; ^4^ 2040 mJ/cm^2^ and 30 s water spraying; ^5^ 2040 mJ/cm^2^ and 30 s 2000 ppm PAA spraying; 5 = best, meat typical 1 = worst, not typical; ^abcd^ mean values in a column with a different letter differ significantly (*p* ≤ 0.05) by Tukey’s test.
